# Self-contamination during doffing of personal protective equipment by healthcare workers to prevent Ebola transmission

**DOI:** 10.1186/s13756-018-0433-y

**Published:** 2018-12-22

**Authors:** Lorna K. P. Suen, Yue Ping Guo, Danny W. K. Tong, Polly H. M. Leung, David Lung, Mandy S. P. Ng, Timothy K. H. Lai, Kiki Y. K. Lo, Cypher H. Au-Yeung, Winnie Yu

**Affiliations:** 1 School of Nursing, The Hong Kong Polytechnic University, Hung Hom, Hong Kong, Special Administrative Region of China, China; 2 Hospital Authority, Hong Kong, Special Administrative Region of China, China; 3 Department of Health Technology and Informatics, The Hong Kong Polytechnic University, Hung Hom, Hong Kong, Special Administrative Region of China, China; 4 Department of Clinical Pathology, Tuen Mun Hospital, Tuen Mun, Hong Kong, Special Administrative Region of China, China; 5 Infectious Disease Centre, Princess Margaret Hospital, Hong Kong, Special Administrative Region of China, China; 6 Institute of Textiles & Clothing, The Hong Kong Polytechnic University, Hung Hom, Kowloon, Hong Kong, Special Administrative Region of China, China

## Abstract

**Background:**

Healthcare workers (HCWs) use personal protective equipment (PPE) in Ebola virus disease (EVD) situations. However, preventing the contamination of HCWs and the environment during PPE removal crucially requires improved strategies. This study aimed to compare the efficacy of three PPE ensembles, namely, Hospital Authority (HA) Standard Ebola PPE set (PPE1), Dupont Tyvek Model, style 1422A (PPE2), and HA isolation gown for routine patient care and performing aerosol-generating procedures (PPE3) to prevent EVD transmission by measuring the degree of contamination of HCWs and the environment.

**Methods:**

A total of 59 participants randomly performed PPE donning and doffing. The trial consisted of PPE donning, applying fluorescent solution on the PPE surface, PPE doffing of participants, and estimation of the degree of contamination as indicated by the number of fluorescent stains on the working clothes and environment. Protocol deviations during PPE donning and doffing were monitored.

**Results:**

PPE2 and PPE3 presented higher contamination risks than PPE1. Environmental contaminations such as those originating from rubbish bin covers, chairs, faucets, and sinks were detected. Procedure deviations were observed during PPE donning and doffing, with PPE1 presenting the lowest overall deviation rate (%) among the three PPE ensembles (*p <* 0.05).

**Conclusion:**

Contamination of the subjects’ working clothes and surrounding environment occurred frequently during PPE doffing. Procedure deviations were observed during PPE donning and doffing. Although PPE1 presented a lower contamination risk than PPE2 and PPE3 during doffing and protocol deviations, the design of PPE1 can still be further improved. Future directions should focus on designing a high-coverage-area PPE with simple ergonomic features and on evaluating the doffing procedure to minimise the risk of recontamination. Regular training for users should be emphasised to minimise protocol deviations, and in turn, guarantee the best protection to HCWs.

**Electronic supplementary material:**

The online version of this article (10.1186/s13756-018-0433-y) contains supplementary material, which is available to authorized users.

## Introduction

Ebola virus disease (EVD) is a severe infectious disease with a high fatality rate of approximately 50% [1]. The virus in the blood and body fluids of a patient can enter another person’s body through skin lesions or mucous membranes of the eyes, nose or mouth. Therefore, health care workers (HCWs) should wear protective gear and adopt strict infection control measures when caring for suspected patients [2, 3].

The EVD outbreak has recently prompted interest in personal protective equipment (PPE) apparel and their use [4]. PPE comprise gowns, gloves, hood, face shield, boots, masks or respirators, which are used to protect HCWs from contact with infectious agents. However, although equipped with protective clothing, HCWs can be contaminated if the PPE apparel is improperly removed [3]. PPE must be removed slowly, deliberately and in the correct sequence to reduce the possibility of self-contamination or exposure to EVD [5].

Several healthcare organisations developed PPE protocols based on the best locally available components. However, HCWs may be hesitant to use a PPE with no empirical validation [4]. Thus, crucial precautions during PPE removal must be determined to effectively protect HCWs [6].

The Hospital Authority (HA) of Hong Kong is a statutory body that manages Hong Kong’s public hospital services [7]. The HA recommends a PPE ensemble with a neck-to-ankle overall without skin exposure to meet the current recommendations of the Centers for Diseases Control and Prevention (CDC) on the PPE to be used by HCWs during management of patients with confirmed EVD [5]. A waterproof hood and a water-resistant gown were designed to cover the head, neck and body of HCWs. Previous studies [8, 9] reported that a water-resistant gown can provide a good physical barrier via preventing the absorption of liquid contaminants, and thus, conferring protection to HCWs who come in contact with body fluids and secretions of patients with EVD. Our previous study has shown that the barrier protection performance and usability of PPEs are affected by the covered area and ergonomic features [8]. However, systematic data on the risk of self-contamination of different PPE types for Ebola prevention remain lacking. In the present study, three types of PPEs, namely, Hospital Authority Standard Ebola PPE set (PPE1), Dupont Tyvek Model, style 1422A (PPE2) and HA isolation gown for routine patient care and performing aerosol-generating procedures (PPE3), were tested. We compared the PPE ensembles used to prevent EVD transmission in terms of protocol deviations during usage and the degree of contamination during doffing.

## Materials and methods

This research was an experimental study of one group using multiple comparisons.

### Study participants

A total of 59 HCWs were recruited for this study. The sample size was determined as previously described by Guo et al. [8], who have examined body-contamination rates and environmental-contamination levels during doffing of different PPE types in accordance with the protocol recommended by the HA. Pregnant females and participants suffering from upper respiratory tract infection and respiratory diseases requiring treatment were excluded.

Among the participants, 57.60% (*n =* 34) were female with an age range of 21–60 years old. The participants were either registered nurses (*n* = 50, 84.80%), advanced practicing *nurses* (*n =* 4, 6.80%), nursing officers (*n =* 3, 5.10%) or nurse educators (*n* = 2, 3.40%). The participants worked in units with high infection risk, including the intensive care unit, emergency department, infection control units and respiratory wards, accounting for 47.50% (*n* = 28), whilst the rest worked in units with relatively low infection risk (i.e., other clinical units apart from the units mentioned above [*n* = 31, 52.50%]). All the participants have not yet worn PPE2 because this ensemble is generally not adopted in local hospitals for HCWs. Participants who are currently working in high-infection -risk units have more opportunities to wear PPE1 and PPE3 in daily practice.

### PPE ensembles under testing

Three PPE ensembles were tested (Additional file 1: Figure S1, Additional file 2: Figure S2, Additional file 3: Figure S3, Additional file 4: Figure S4, Additional file 5: Figure S5, Additional file 6: Figure S6). (1) HA standard Ebola PPE set (PPE1) is a neck-to-ankle overall with an overlying water-resistant gown (Halyard, AAMI Level 4 Liquid Barrier Standard), double and long nitrate gloves, boots, hood, disposable face shield and N95 respirator. A bow was tied at the lateral of the waist to minimise the risk of front contamination. (2) DuPont™ Tyvek®, Model 1422A (PPE2) is commonly adopted in clinical settings to prevent Ebola transmission in countries, such as the US [10, 11] and South Korea [3]. Its protective clothing is also fluid resistant, but the design is a one-piece head-to-ankle overall with a zipper on the front. The whole outfit includes double gloves, boots, disposable face shield and an N95 respirator. A plastic apron was used to cover up the front zipper before use. (3) PPE3 is a HA isolation gown (Medicom®) for routine patient care and performing aerosol-generating procedures. PPE3 was selected as the reference PPE in the present study. A commercially available pure cotton surgical scrub suit (upper and lower working clothes) was worn inside the individual PPE ensembles during testing. Participants were free to select the appropriate size of gowns and gloves and the known best-fitted respirator model (3 M 1860, 1860s and 1870). Table 1 shows the comparison of the three PPE ensembles.

**Table 1:** Comparison of the three PPE ensembles

Hospital Authority Standard Ebola PPE set (PPE1)	DuPont™ Tyvek®, Model 1422A (PPE2)	Hospital Authority isolation gown for routine patient care and performing aerosol-generating procedures (PPE 3) [As a reference]
Neck-to-ankle outfit	Head-to-ankle coverall	Neck-to-ankle outfit
N95 respirator	N95 respirator	N95 respirator
Hood (Kimberly Clark, model no. 25797)	Hood with elasticated facial opening	No hood
Disposable face shield	Disposable face shield	Disposable face shield
MICROCOOL Breathable High Performance Surgical Gown (Halyard, AAMI Level 4 Liquid Barrier Standard)^a^	Tyvek, a brand of flash spun high-density polyethylene fibers, a synthetic material. Apparel with elasticated wrists and ankles.	Water resistant isolation gown (Medicom®)
No zipper; the bow of the water-resistant gown is tied at the lateral side of the waist.	Zipper along the center front of the coverall, covered by a plastic apron.	No zipper; the bow of the water-resistant gown is tied at the lateral side of the waist.
Boots	Boots	Shoes
Double, long nitrate gloves	Double, long nitrate gloves	Single, latex gloves

^a^AAMI: The Association for the Advancement of Medical Instrumentation

### Procedures

Data collection was performed in an air-conditioned room with an average temperature of 23 °C ± 2 °C and a relative humidity of 60% ± 3%. Information about the purpose and procedures of the study was provided to the participants, and written consent was obtained prior to the study.

The participants’ socio-demographic data, including gender, age, educational background, specialty, working units and clinical experience, were collected. Each subject received a 30 min briefing from a trained research personnel. The donning and doffing procedures for PPE1 and PPE3 were designed based on the recommendations by the HA, whilst the World Health Organisation (WHO) protocol was followed for PPE2 doffing [12]. On the testing day, the participants watched a video about donning and doffing of the PPE ensembles to familiarise themselves with the procedures. The total duration for donning and doffing of PPEs in the videos was 8.74, 10.68 and 4.59 min for PPE1, PPE2 and PPE3, respectively. Posters related to donning and doffing procedures were pinned up in the venue. Participants with long hair were asked to tie up their mane. Watch and jewellery were removed to minimise the risk of exposure during the procedures. Afterwards, the participants donned and doffed the three PPEs in a random order as decided by a computer-generated randomised table.

The experiment was sequentially conducted in three areas. Area A was the ‘clean zone’, where the participants donned the working clothes and clean PPE ensemble in front of a mirror. Area B was the ‘preparation zone’, where the PPE of the participants was contaminated with a fluorescent solution (UV GERM Hygiene Spray, Glow Tec Ltd., London, England) that mimics contaminated bodily fluids or secretions spread via contact route. Fluorescent solution was sprayed onto the face shield, two upper limb/gloves and anterior surfaces of the gown at a distance of 60 cm from the participants, which represents the length of a stethoscope, simulating the usual working distance between a patient and an HCW [8], with an average of 1.99 g fluorescent solution/per stroke [9]. This value was determined using an electronic analytical balance with a precision of 0.1 g (NJW-3000, Xiangxin, Taipei, Taiwan) via obtaining the average of 20 trial cases. A standard of three strokes was sprayed on each body part with a total of 12 strokes made for each case. The weight of the splash in 1 stroke was 1.99 g in this study when the density of the solution was assumed as 1. Area C is the ‘degown and test zone’, wherein the participants were required to doff the PPE. A video camera with a high-density capability was set up for subsequent evaluation of protocol deviations during donning and doffing. Protocol deviations are defined as accidental or noncompliance with the donning and doffing procedures of the PPEs under testing. The performance of the participants was monitored using a checklist. The participants were notified immediately of any deviation being committed. For evaluation, all protocol deviations were recorded. The participants were timed and videotaped whilst donning and doffing the PPE. The timer was stopped when the participants removed the final item of the protective clothing.

During the procedures, hand washing with liquid soap and water was performed according to the procedures of individual PPEs. Immediately after doffing, the participants were scanned for the presence of fluorescent solution. The participants’ body (hair and head, face, anterior/posterior neck, left/right arms, hands or wrists, upper/lower working clothes and clogs) and the surrounding environment (rubbish bin cover, chair, faucet and sink) were examined using an ultraviolet lamp (CheckPoint, 220–240 V/50 Hz; Glow Tec Ltd., London, England) under dim light. Areas of contamination were counted and measured in square centimetres, and the fluorescent patches of different sizes were counted. Contaminated stains were defined as either small- (≤1cm^2^), medium- (1cm^2^ to <3cm^2^), large- (≥3cm^2^ to 5cm^2^) or extra-large patch (≥5cm^2^) [8, 13, 14]. The number of contaminated patches and the time consumed by the participants during donning and doffing were recorded. The environment was thoroughly cleaned, and the areas were rechecked for any contamination with the ultraviolet lamp before the next trial. A 15 min break was given, and water was provided to the participants before testing the next PPE to prevent fatigue, which may affect performance.

### Statistical analyses

All data were analysed with IBM SPSS Statistics 23. Descriptive statistics were used for all independent variables, including the subjects’ age, gender, position, working specialty and clinical experience in years. The contamination sites among the PPEs were compared with χ^2^ or Fisher’s exact test as required. The degree of contamination during doffing and the time for PPE donning and doffing were compared using one-way ANOVA. Protocol deviations were expressed as the deviation rate (%). All differences reported were considered significant at the *p* < 0.05 level.

## Results

### Degree of contamination during doffing

Contamination by small patches on the working clothes of the wearers occurred less frequently during PPE1 removal than during PPE2 and PPE3 removal (median: 5.00 versus 7.00 versus 7.00, *p <* 0.05). The degree of contamination by large patches occurred less frequently during the removal of PPE1 and PPE2 than during the removal of PPE3 (median: 1.00 versus 1.00 versus 2.00, *p <* 0.05). No significant difference in medium-contaminated patches and number/area of extra-large contaminated patches was observed among the three PPEs. The older staff (aged 41–50 and 51–60 years old) featured significantly less-small contaminated patches than that of the younger staff (aged 21–30 and 31–40 years old) (*p <* 0.05). No gender differences were observed in the degree of contamination among the ensembles under testing. Moreover, HCWs working in units with relatively low infection risk showed significantly less small contaminated patches than those working in units with high infection risk (median: 5.00 versus 8.00, *p <* 0.001).

### Contamination sites during doffing

Table 2 shows the distribution of contaminated sites on the body and the surrounding environment during doffing. The overall contamination of PPE1 during doffing is relatively less than those of PPE2 and PPE3, as indicated by either small- or extra-large contaminated patches. PPE2 is relatively more heavily contaminated than PPE1 during doffing in sites, such as hands and wrists, working clothes and the environment (chair). Meanwhile, PPE3 is more easily contaminated in the neck regions, clogs and arms than PPE1.

**Table 2:** Sites of contamination during doffing of personal protective equipment

	Small sized contaminated patches (< 1 cm ^2^), median				Extra large sized contaminated patches (≥ 5 cm^2^), median			
Location	PPE1	PPE2	PPE3	*p*-value	PPE1	PPE2	PPE3	*p*-value
Hair and head	1.00	2.00	2.50	0.68	0.00	17.00	0.00	N/A
Face	1.00	4.00	2.00	0.602	0.00	0.00	8.00	N/A
Neck (anterior)	2.50	5.00	11.00	0.095	0.00	0.00	24.00	N/A
Neck (posterior)	2.00	1.00	18.50	0.824	0.00	0.00	0.00	N/A
Arms (right)	3.50	1.00	4.00	0.414	0.00	0.00	28.00	N/A
Arms (left)	2.00	2.00	1.00	0.909	0.00	0.00	49.00	N/A
Hands or wrists	1.00	1.00	6.00	0.414	8.00	61.00	0.00	N/A
Working clothes (upper)	8.50	9.00	7.00	0.997	21.00	48.50	42.00	0.690
Working clothes (lower)	2.00	2.50	6.00	0.111	12.00	46.00	17.50	0.276
Clogs	3.00	5.00	13.50	< 0.001*	121.00	55.00	133.00	0.397
Environment (rubbish bin cover)	2.00	7.00	2.50	0.254	20.00	14.00	23.00	0.737
Environment (chair)	3.00	6.50	2.00	0.053	0.00	36.00	0.00	N/A
Faucet	2.00	2.00	1.50	0.659	0.00	16.00	14.00	N/A
Sink	12.50	14.00	10.00	0.072	75.50	66.50	44.00	0.649
Overall	5.00	7.00	7.00	0.05*	39.00	43.00	47.00	< 0.001*

*significant *p* values

N/A: There are fewer than two groups for the dependent variables, so no inferential statistics are computed using ANOVA

PPE1: Hospital Authority Standard Ebola PPE set

PPE2: DuPont™ Tyvek®, Model 1422A

PPE3: Hospital Authority isolation gown for routine patient care and performing aerosol-generating procedures

### Donning and doffing protocol deviations

Procedure deviations during donning and doffing of the PPEs were observed, with PPE1 exhibiting the lowest overall deviation rate among the three PPE ensembles during doffing (2.95, 9.48 and 3.52% for PPE1, PPE2 and PPE3, respectively). The top three highest deviation percentages in each type of PPE are in bold (Table 3). Participants working in units with high infection risk presented significantly lower deviation rate than those working in units with low infection risk during donning of PPE1 and PPE2, but no significant differences in the deviation rates can be observed among the groups during doffing of the three PPEs.

**Table 3:** The deviation rate (%) during donning and doffing of personal protective equipment

Steps	Donning of PPE1		Donning of PPE2		Donning of PPE3	
	Procedure	Error (%)	Procedure	Error (%)	Procedure	Error (%)
1.	Visually inspect the PPE ensemble	0.00	Visually inspect the PPE ensemble	0.00	Visually inspect the PPE ensemble	0.00
2.	Perform hand hygiene	1.67	Perform hand hygiene	6.67	Perform hand hygiene	1.67
3.	Put on N95 respirator and perform fit test	3.33	Put on inner gloves	1.67	Put on N95 respirator and perform fit test	3.33
4.	Perform hand hygiene	5.00	Put on coverall & inspect for integrity	**8.33**	Perform hand hygiene	**6.67**
5.	Put on hood	**20.00**	Put on rubber boots	1.67	Put on cap	3.33
6.	Put on full face-shield	**11.67**	Put on N95 respirator and perform fit test	3.33	Put on full face-shield	**6.67**
7.	Put on gown	8.33	Put on face shield	**15.00**	Put on gown	1.67
8.	Perform hand hygiene	**13.33**	Put on hood	3.33	Perform hand hygiene	**10.00**
9.	Put on boots	0.00	Put on outer apron	**13.33**	Put on gloves	0.00
10.	Perform hand hygiene	3.33	Put on outer gloves	6.67		
11.	Put on gloves	0.00				
	**Overall deviation rate**	6.06		6.00		3.70
	**Doffing of PPE1**		**Doffing of PPE2**		**Doffing of PPE3**	
1.	Remove gloves	1.67	Disinfect outer gloves	**13.33**	Remove gloves	0.00
2.	Perform hand hygiene	0.00	Remove apron	11.67	Perform hand hygiene	1.67
3.	Remove gown	5.00	Disinfect outer gloves	**13.33**	Remove gown	**6.67**
4.	Perform hand hygiene	0.00	Remove hood	8.33	Perform hand hygiene	1.67
6.	Put clogs on the floor	5.00	Disinfect outer gloves	6.67	Remove full face-shield	**10.00**
7.	Take off the boots and put on clogs	**10.00**	Remove coverall and outer gloves together	**58.33**	Remove cap	**8.33**
8.	Perform hand hygiene	1.67	Disinfect inner gloves	1.67	Perform hand hygiene	0.00
	Remove full face-shield	**6.67**	Remove face shield	11.67	Remove N95 respirator	3.33
9.	Perform hand hygiene	0.00	Disinfect inner gloves	3.33	Perform Hand Hygiene	0.00
10.	Remove hood	**5.00**	Remove N95 respirator	3.33		
11.	Perform hand hygiene	0.00	Disinfect inner gloves	1.67		
12.	Remove N95 respirator	3.33	Put clogs on the floor	3.33		
13.	Perform hand hygiene	0.00	Remove boots and put on clogs	8.33		
14.			Disinfect inner gloves	5.00		
15.			Remove inner gloves	1.67		
16.			Perform hand hygiene	0.00		
	**Overall deviation rate (%)**	2.95		9.48		3.52

The top three highest deviation percentages in each type of PPE were bold

PPE1:Hospital Authority Standard Ebola PPE set

PPE2: DuPont™ Tyvek®, Model 1422A

PPE3: Hospital Authority isolation gown for routine patient care and performing aerosol-generating procedures

### Timing for PPE donning and doffing

On the average, the participants used the longest time to don and doff PPE2, followed by PPE1 and PPE3. Donning PPE1, PPE2 and PPE3 required an average of 6.61 (range: 4.00–14.41 min), 7.29 (range: 4.48–14.52 min) and 3.28 min (range: 1.34–7.36 min), respectively, whereas doffing required 6.97 (range: 3.28–14.33 min), 10.37 (range: 5.43–23.53 min) and 4.42 min (range: 2.08–12.23). The HCWs working in units with relatively low infection risk showed significantly longer donning time when wearing PPE2 and PPE3 than those working in units with high infection risk. However, no significant differences were observed in the time used for doffing all PPEs under testing (Table 4).

**Table 4:** Time (in minutes) required for donning and doffing of personal protective equipment

	All participants Mean (sd) *n* = 59	High-risk group Mean (sd) *n* = 28	Low-risk group Mean (sd) *n* = 31	*p*-value # (t-test, high risk vs low risk)
Time required for donning (min)				
PPE1	6.59 (1.67)	6.19 (1.13)	6.94 (1.99)	0.086
PPE2	7.26 (2.06)	6.63 (1.77)	7.83 (2.16)	< 0.05*
PPE3	3.28 (1.15)	2.90 (0.98)	3.63 (1.25)	< 0.05*
	*P* < 0.001*			
Time required for doffing (min)				
PPE1	6.95 (2.59)	6.79 (2.42)	7.10 (2.77)	0.659
PPE2	10.31 (3.93)	10.24 (3.85)	10.38 (4.07)	0.893
PPE3	4.44 (1.87)	4.30 (1.38)	4.56 (2.23)	0.594
	*P* < 0.001*			

#t-test, participants working in units with higher infection risk versus working with lower infection risk

*significant *p* values PPE1: Hospital Authority Standard Ebola PPE set PPE2: DuPont™ Tyvek®, Model 1422A

PPE3: Hospital Authority isolation gown for routine patient care and performing aerosol-generating procedures

## Discussion

### Self-contamination during doffing

Our study demonstrated considerable self-contamination during doffing. This result raised concerns on pathogen contamination of the skin or clothes of HCWs during PPE removal, which may result in self-inoculation and spread of the virus to patients and other HCWs through contaminated body fluids, including blood, urine, vomitus and stool. Gastrointestinal fluid losses of patients with EVD can be massive (5–10 L/day), droplet dispersion can be greater than 10 ft. and serum viral loads of dying patients with EVD can reach 10 billion copies/mL [15]. Given that no licensed vaccines nor proven effective antiviral therapies for EVD are currently available, PPE plays a crucial role in mitigating the risk of HCW exposure to contaminated body fluids in the care of patients with EVD [16].

The frequent occurrence of self-contamination during PPE doffing is also consistent with the findings of previous studies [6–8, 13–21]. The most likely contaminated areas include the neck, hands and fingers, arms and wrists and face [14, 17]. A study conducted in South Korea that estimated the degree of contamination during PPE doffing of HCWs reported that the most vulnerable processes comprise the removal of the respirator, shoe cover and hood [3].

The current study indicated that contamination of the working clothes occurred less frequently during PPE1 removal than during the removal of PPE2 and PPE3, which may be due to the ergonomic features of individual PPEs under testing. PPE1 consists of a neck-to-ankle outfit and includes a hood covering the neck. PPE2 is a head-to-ankle overall, and is a PPE ensemble frequently used in overseas settings to prevent Ebola transmission [3, 10, 11]. However, PPE removal is complicated because of the head-to-ankle, one-piece design and the elastication in the facial opening, wrists and ankles. HCWs have to take off the hood, unzip the front zipper, remove the overall and outer gloves together and place the trousers on the chair, thereby resulting in easy contamination of the hair and head, hands, working clothes, clogs and chair [17]. The elasticated one-piece coverall hood creates a potential contamination risk because the elastic contracts and pulls the outer part of the hood inwards and towards the participants’ hair and neck during removal [22]. The zipper and its flap are also placed along the PPE2 centre front. Therefore, a plastic apron is worn to minimise the risk of body fluids being trapped in the zipper region. Herlihey et al. [22] also reported that when the subjects unzip the coverall, the zipper is stuck in the surrounding fabric or the gloves are stuck to the adhesive of the PPE, while unsealing the flaps covering the zipper results in ripping [22].

The WHO protocol requires the overall to be removed from top to bottom, followed by the removal of outer gloves whilst pulling the arms out of the sleeves of the overall. Special caution is needed to prevent self-contamination. PPE3 is recommended by HA for routine patient care, in which the neck, lower part of the legs and shoes are incompletely covered. Compared with PPE 1, additional sites, including the neck, arms, working clothes and clogs, were heavily contaminated when wearing PPE3 because it cannot provide adequate protection for HCWs caring for patients with EVD. These contaminated regions may be caused by self-contamination during doffing and contamination when the fluorescent solution was sprayed. Considering the possible underclothing contamination during doffing, the working clothes worn under the PPE ensembles should be frequently changed, especially when contamination is suspected.

During PPE1 or PPE2 doffing, the participants have to wear the clean clogs after removing their boots. However, the clogs may be possibly contaminated by the gowns or the environment in some cases. Hence, using footwear covers is an unideal option. During boot cover removal, HCWs struggle to balance their legs in the air [20]. Shoe covers are also difficult to doff, thereby often requiring assistance and increasing the risk of cross-contamination among workers [22].

The CDC and WHO recommend the use of double gloving with at least the outer pair possessing an extended cuff that reaches beyond the wrist [6] to decrease the incidence of hand contamination and provide improved protection for HCWs during PPE removal [16, 23]. Although double gloving is incorporated into the protocols for PPE use, the removal of the outer and inner gloves should be done with caution, followed by proper hand hygiene.

Previous studies defined contamination as small fluorescent stains (<1cm^2^) and large patches (>1cm^2^) [8, 13, 14] and revealed that fluorescent stain sizes are affected during gown removal [8]. In the present study, a precise estimation of the contaminated regions was performed in terms of the size of patches, that is, small (≤1cm^2^), medium (1cm^2^ to <3cm^2^), large (≥1cm^2^ to 5cm^2^), or extra large (≥5cm^2^). The stain sizes can be associated with either inadequate PPE coverage or because of self-contamination during PPE removal. For example, PPE3 cannot fully cover the neck of the participants, which resulted in many small or extra-large patches in the anterior and posterior neck region after spraying of the fluorescent solution onto the face shield and anterior surfaces of the gown. Meanwhile, PPE2 offers a high coverage area during fluorescent solution spraying. However, the hair/head, hands or wrists of the participants were heavily contaminated with extra-large patches during PPE removal. Similarly, medium-sized patch contamination can be due to either the PPE design or self-contamination. Therefore, a PPE with a high coverage area and simple ergonomic features that can minimise the risk of recontamination during doffing should be designed.

In this study, the older staff showed significantly less small-sized contaminated patches on their working clothes than the younger staff. This result may be due to the additional cautiousness of the older staff, whilst working than the younger staff. However, this finding cannot be generalised because of the low number of older staff (*n =* 4) who participated in this study.

### Environmental contamination

In addition to self-contamination of HCWs during PPE doffing, environmental contaminations, such as those in the lid of the rubbish bin, chair, faucet and sink, were observed. Human-to-human transmission of EVD is also possible via indirect contact with the environment contaminated with such fluids [24]. The virus can survive for several hours on dry surfaces, such as doorknobs and countertops, to several days at room temperature in body fluids, such as blood [25]; virus-positive samples were still observed 7 days post-mortem [16].

Considering that hand hygiene methods using alcohol hand sanitiser fail to remove the fluorescent solution, handwashing with soap and water was performed by the participants. Thus, the sink may be contaminated because of handwashing, and the working clothes that came in contact with the sink may be contaminated because of the repeated handwashing. These results suggested that the height and width of the sink must be at a good working level of HCWs to prevent self-contamination during handwashing. Although alcohol gel is commonly used nowadays during PPE donning/doffing, hand cleansing with soap and water is recommended in cases of visible contamination in various situations, such as when areas are contaminated by vomitus, respiratory secretions, or fecal matter. Discarding used PPEs should be given much attention because of frequent contamination of rubbish bin covers [13].

### Protocol deviations and importance of training

Deviations of the donning procedure may increase the risk of self-contamination whilst doffing [20]. Although the participants watched a video on PPE donning and doffing to familiarise themselves with the steps on the day of testing, they can also refer to the posters related to the procedures available in the venue. PPE1 exhibited the lowest overall deviation rate among the three PPE ensembles during doffing (2.95, 9.48 and 3.52% for PPE1, PPE2 and PPE3, respectively). This finding was expected because of the complexity of PPE2, as described above. The highest deviation rate (58.33%) was observed during the simultaneous removal of the overall and outer gloves in PPE2. As mentioned above, this result agrees with the WHO protocol for doffing overall [12]. This protocol requires the participants to remove the inner gloves, which were covered by the coverall. This procedure is difficult for many participants because they can only ‘feel’ the inner gloves during removal and cannot see them. Therefore, several participants cannot remove the overall and outer gloves together, or in certain situations, they removed both the inner and outer gloves simultaneously. Apart from the emphasis on regular training for HCWs to perform the procedure smoothly, the doffing procedure should be evaluated to increase its practicability for the users.

Being an international aviation hub, Hong Kong is frequently visited by travellers from all around the world. Moreover, contacts between Mainland China and African countries are becoming increasingly frequent. Although most HCWs in Hong Kong possess inadequate experience in handling EVD cases, providing regular training for HCWs is necessary to fill the gap between the desired PPE performance and actual practice. Contamination errors caused by unfamiliarity with the procedures, complexity with PPE ensembles and unconscious habits can be prevented through repeated practice and training. Evidence shows that traditional learning methods (e.g., watching educational videos and learning PPE guidelines) are inferior to immersive learning methods, including audio-visual devices and active learning involvement using simulation training that includes feedback on performance for clinical management of EVD cases, in guiding the PPE procedures [3, 17, 26, 27].

On the average, participants used the longest time for donning and doffing PPE2, followed by PPE1 and PPE3. A study reported that HCWs may show poor compliance with proper PPE removal protocol because of time constraints [28]. The most time-consuming processes include removing the shoe covers, putting on gloves and removing the outer gloves [3]. Thus, a short duration of doffing PPE is important for the faultless completion of removal protocol. Familiarisation of the HCWs with the procedures via frequent training and improved ergonomic features is necessary for the PPE design not only to prevent HCWs from self-contamination but also to shorten PPE donning and doffing time.

### Limitations of study

This study has several limitations. Results showed the possibility of Hawthorne effect because the participants knew that they were being observed during the study. Therefore, compared with previous findings, real-life contamination rates or protocol deviations can be poorer than the findings presented in this study. Result generalisation is limited given the small number of participants, most of which are relatively young staff. A larger sample size with a better balance of staff seniority than that of the present study should be considered in future trials to evaluate whether clinical experiences influence the PPE performance.

The fluorescent solution used in this study was intended to mimic the mechanical effects of body fluids or secretions of patients with EVD. Although this method can provide a visualisation of contamination, it cannot provide information about viral load and shows no response to alcohol-based hand sanitiser, similar to EVD. Future studies may consider using surrogate viruses, such as MS2 (a surrogate for non-enveloped human viruses) and bacteriophage ϕ6 (a surrogate for enveloped viruses such as Ebola) to allow researchers to obtain quantitative data on virus transfer events and risks to HCWs without exposing participants to the risk of infection [16, 19, 23].

## Conclusion

Our study demonstrated considerable self-contamination during PPE doffing of HCWs. Use of the head-to-ankle one-piece overall (PPE2) may result in a higher contamination risk than that of the neck-to-ankle outfit with a hood covering the neck (PPE1). Environmental contaminations, such as those in rubbish bin cover, chair, faucet and sink were detected. Procedure deviations during donning and doffing of the PPEs were observed, with PPE1 exhibiting the lowest overall deviation rate (%) among the three PPE ensembles during doffing. Although PPE1 showed the best performance in terms of low risk of self-contamination compared with PPE2 and PPE3 during doffing and protocol deviations, the design of PPE1 can be still further improved. Future directions should focus on designing a PPE with a high coverage area and simple ergonomic features. Evaluating the doffing procedure is also necessary to minimise the risk of recontamination during doffing. Regular training for users should be emphasised to minimise protocol deviations and in turn guarantee the best protection of HCWs.

## Additional files

Additional file 1: **Figure S1.** Hospital Authority Standard Ebola PPE set (PPE1). (JPG 1653 kb)

Additional file 2: **Figure S2.** DuPont™ Tyvek®, Model 1422A (PPE2). (JPG 740 kb)

Additional file 3: **Figure S3.** Hospital Authority isolation gown for routine patient care and performing aerosol-generating procedures (PPE 3). (JPG 1800 kb)

Additional file 4: **Figure S4.** After putting on hood and full face-shield (PPE1). (JPG 1630 kb)

Additional file 5: **Figure S5**. After wearing the outer plastic apron (PPE2). (JPG 1964 kb)

Additional file 6: **Figure S6.** After putting on cap and full face-shield (PPE3). (JPG 886 kb)

## Abbreviations

CDC: Centers for disease control and prevention
EVD: Ebola virus disease
HA: Hospital Authority
HCWs: Health care workers
PPE: Personal protective equipment
WHO: World Health Organisation
