# Supplementary figures and images for: Self-contamination during doffing of personal protective equipment by healthcare workers to prevent Ebola transmission
Source: Antimicrob Resist Infect Control. 2018 Dec 22;7:157. doi: 10.1186/s13756-018-0433-y (PMC6303998; doi:10.1186/s13756-018-0433-y)

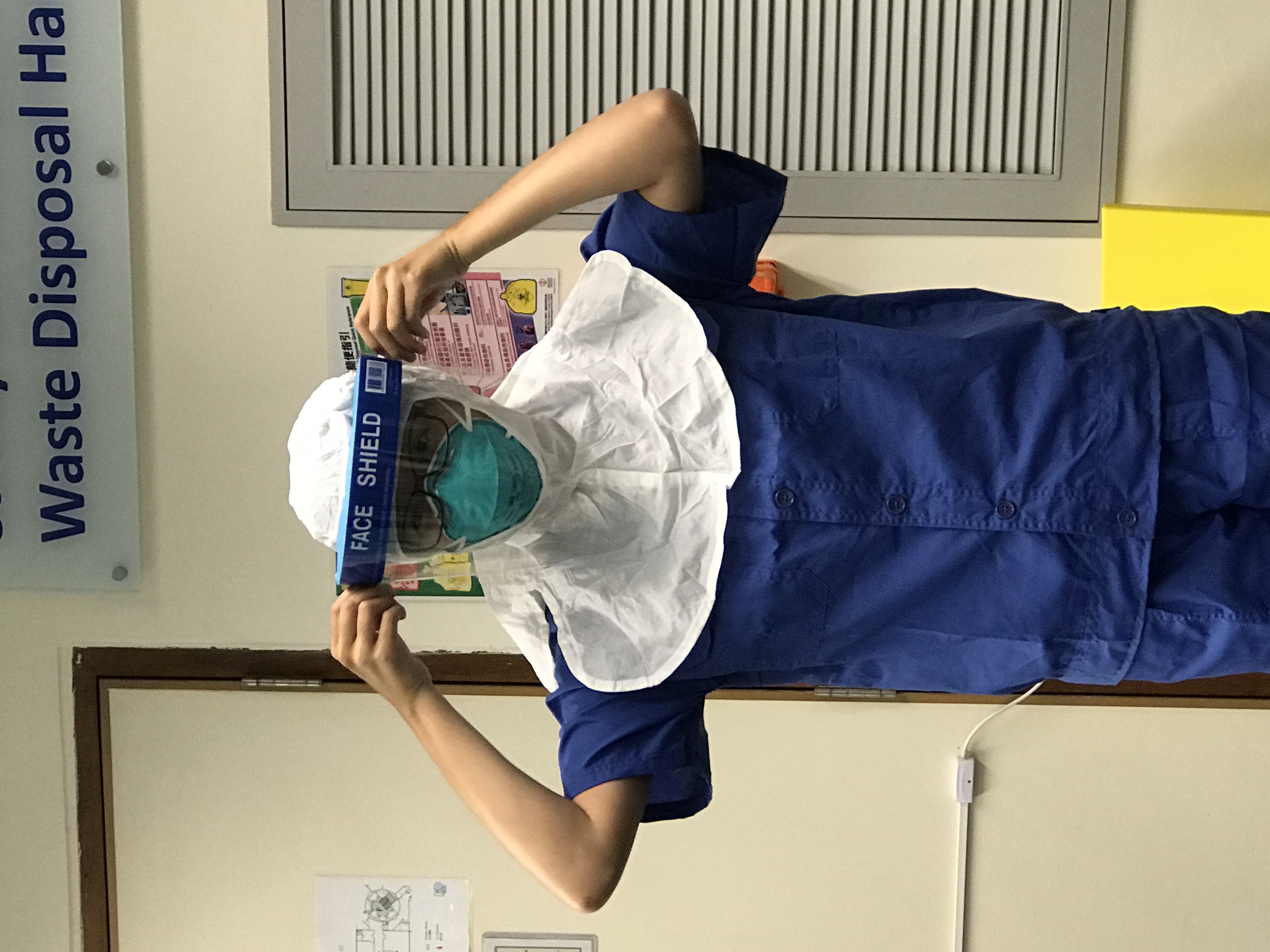

Supplement: Supplementary file 1 — Figure S1. Hospital Authority Standard Ebola PPE set (PPE1). (JPG 1653 kb) [file 13756_2018_433_MOESM1_ESM.jpg]

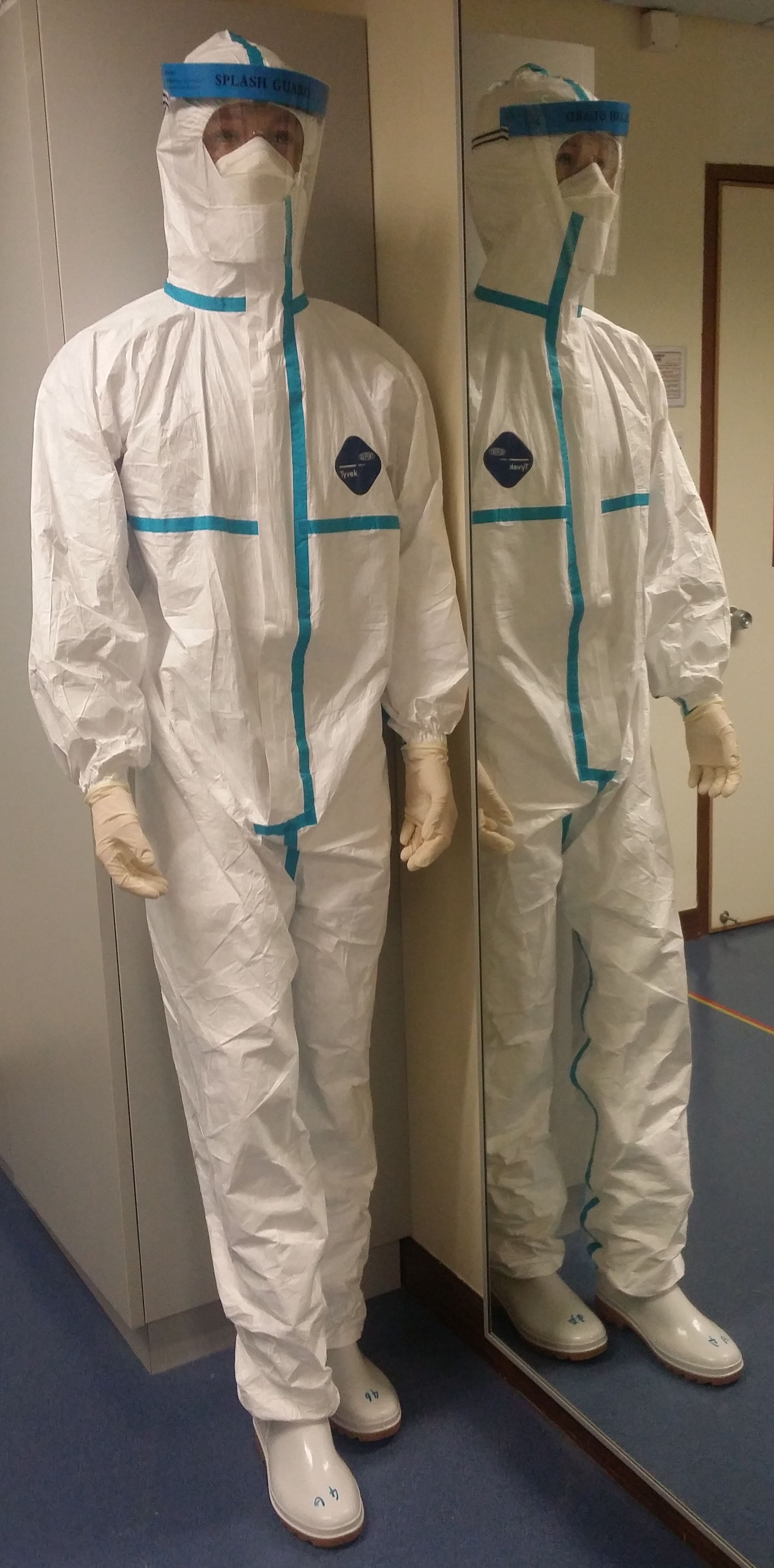

Supplement: Supplementary file 2 — Figure S2. DuPont™ Tyvek®, Model 1422A (PPE2). (JPG 740 kb) [file 13756_2018_433_MOESM2_ESM.jpg]

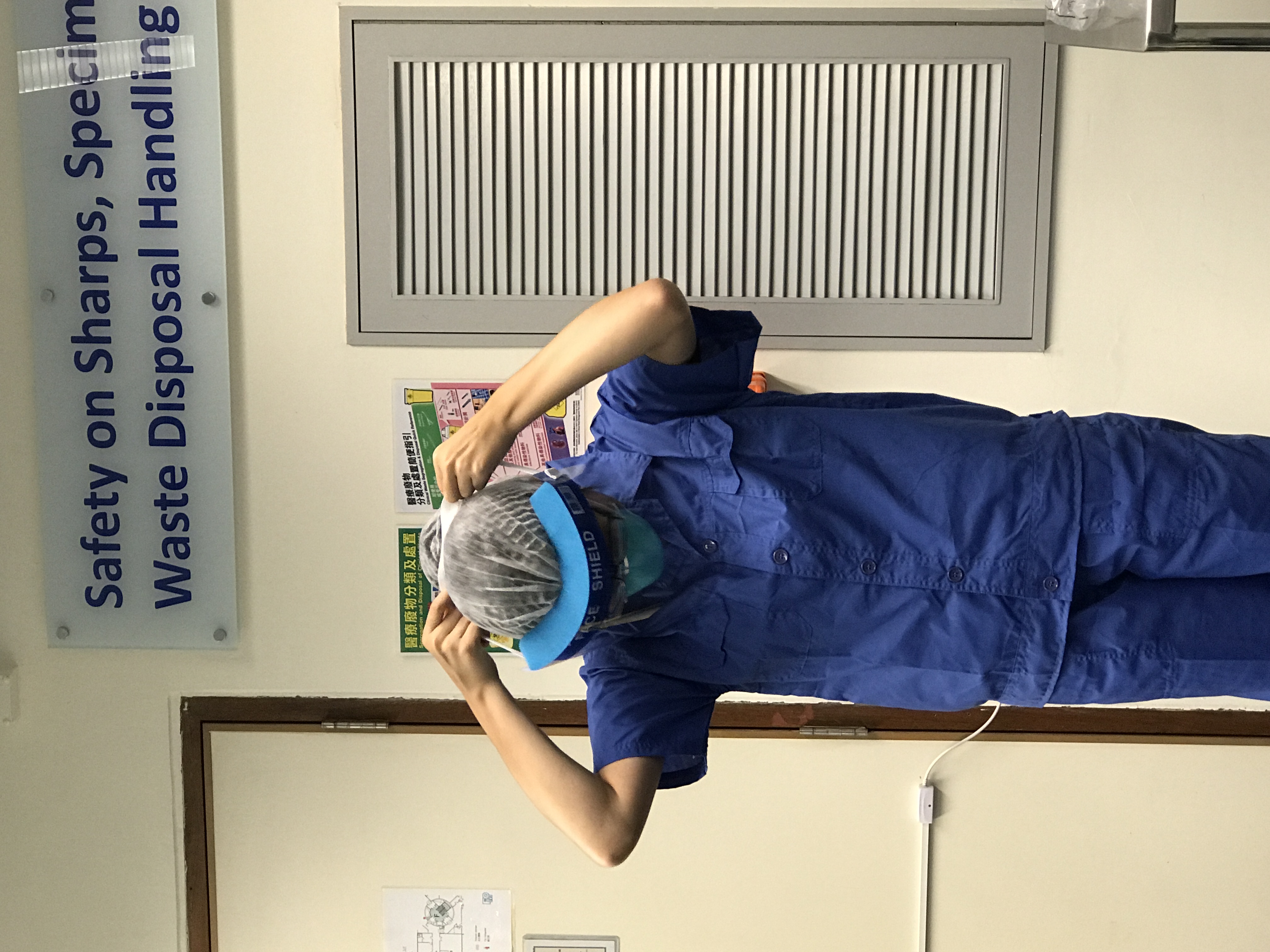

Supplement: Supplementary file 3 — Figure S3. Hospital Authority isolation gown for routine patient care and performing aerosol-generating procedures (PPE 3). (JPG 1800 kb) [file 13756_2018_433_MOESM3_ESM.jpg]

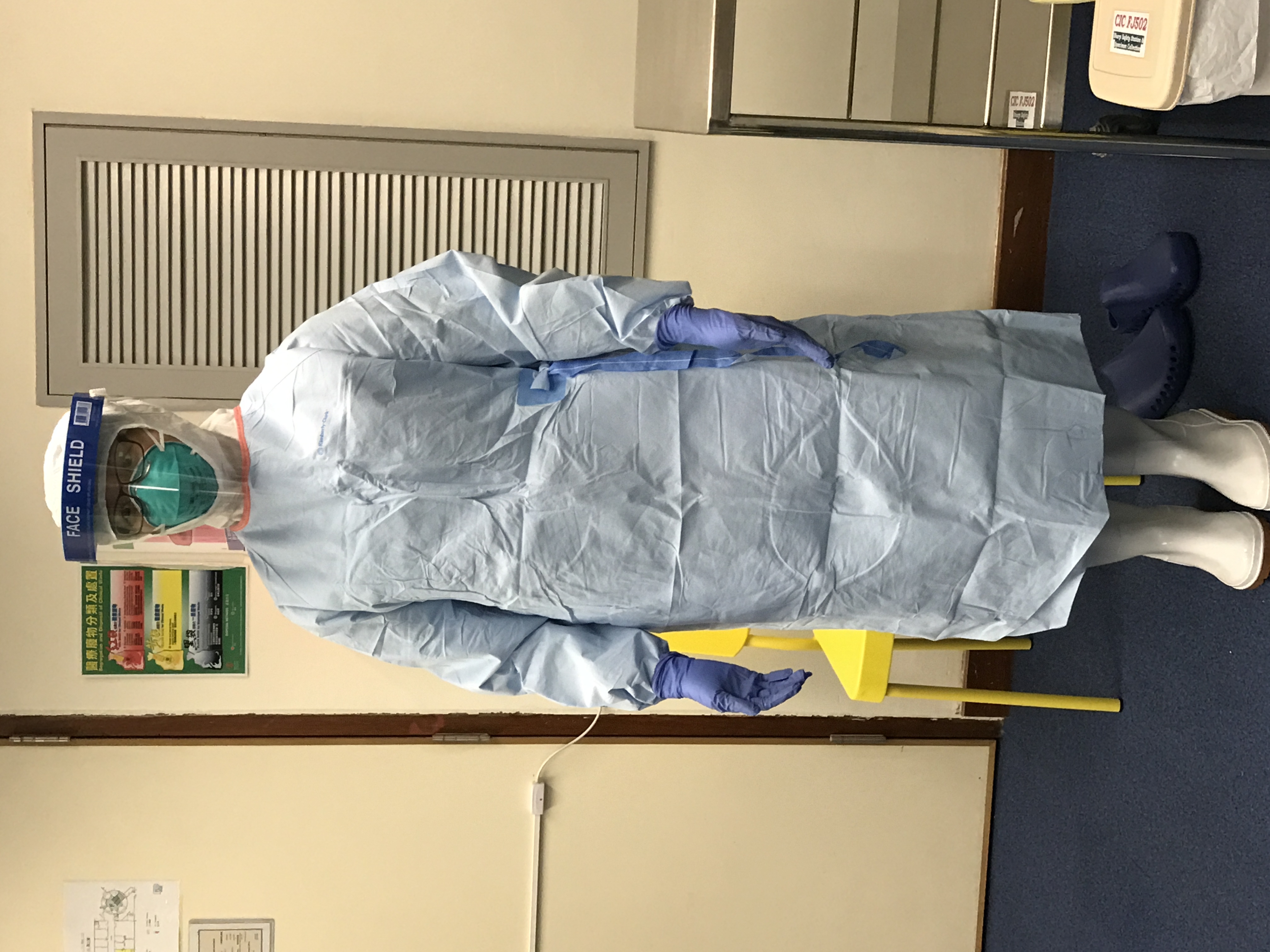

Supplement: Supplementary file 4 — Figure S4. After putting on hood and full face-shield (PPE1). (JPG 1630 kb) [file 13756_2018_433_MOESM4_ESM.jpg]

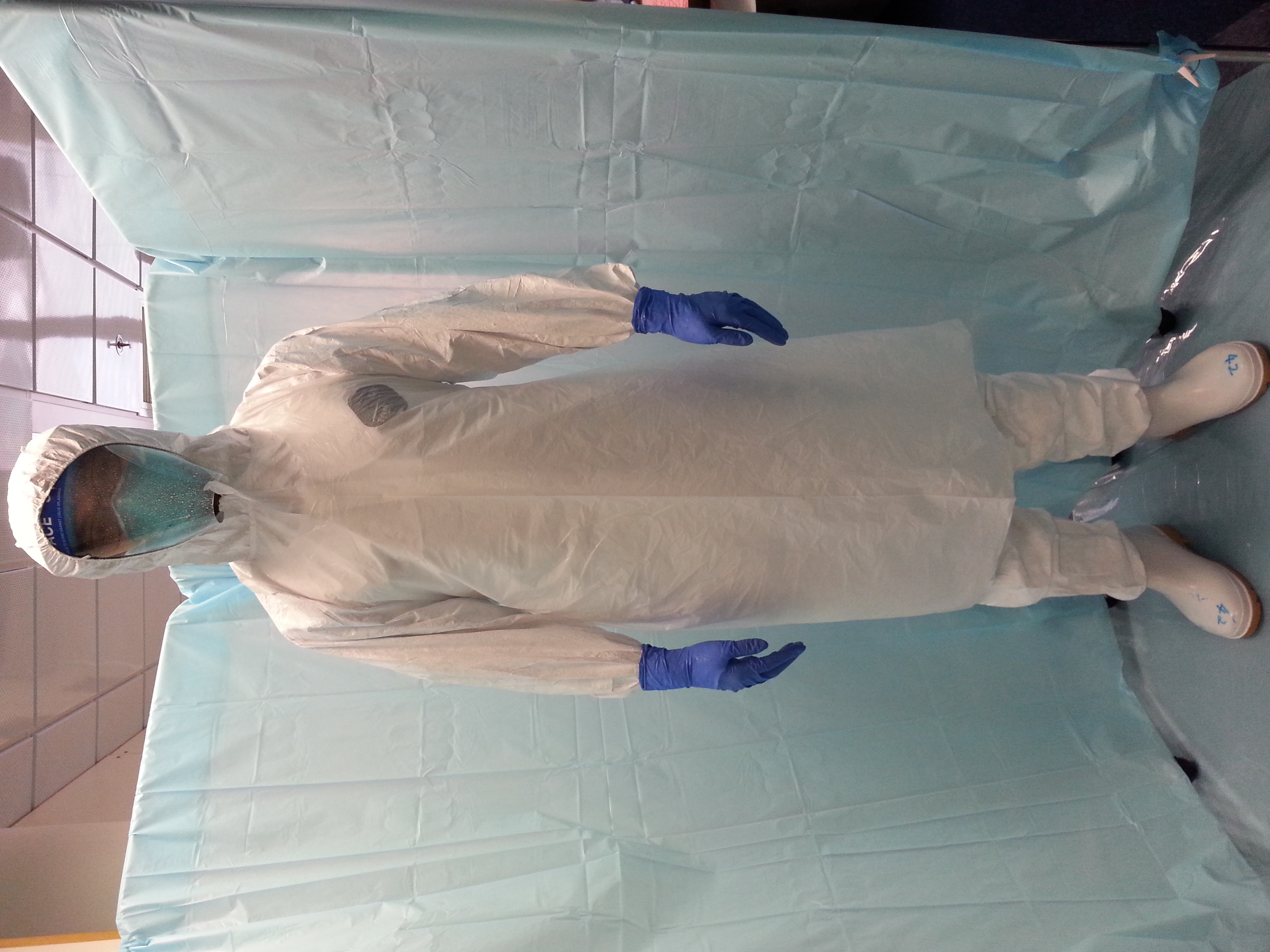

Supplement: Supplementary file 5 — Figure S5. After wearing the outer plastic apron (PPE2). (JPG 1964 kb) [file 13756_2018_433_MOESM5_ESM.jpg]

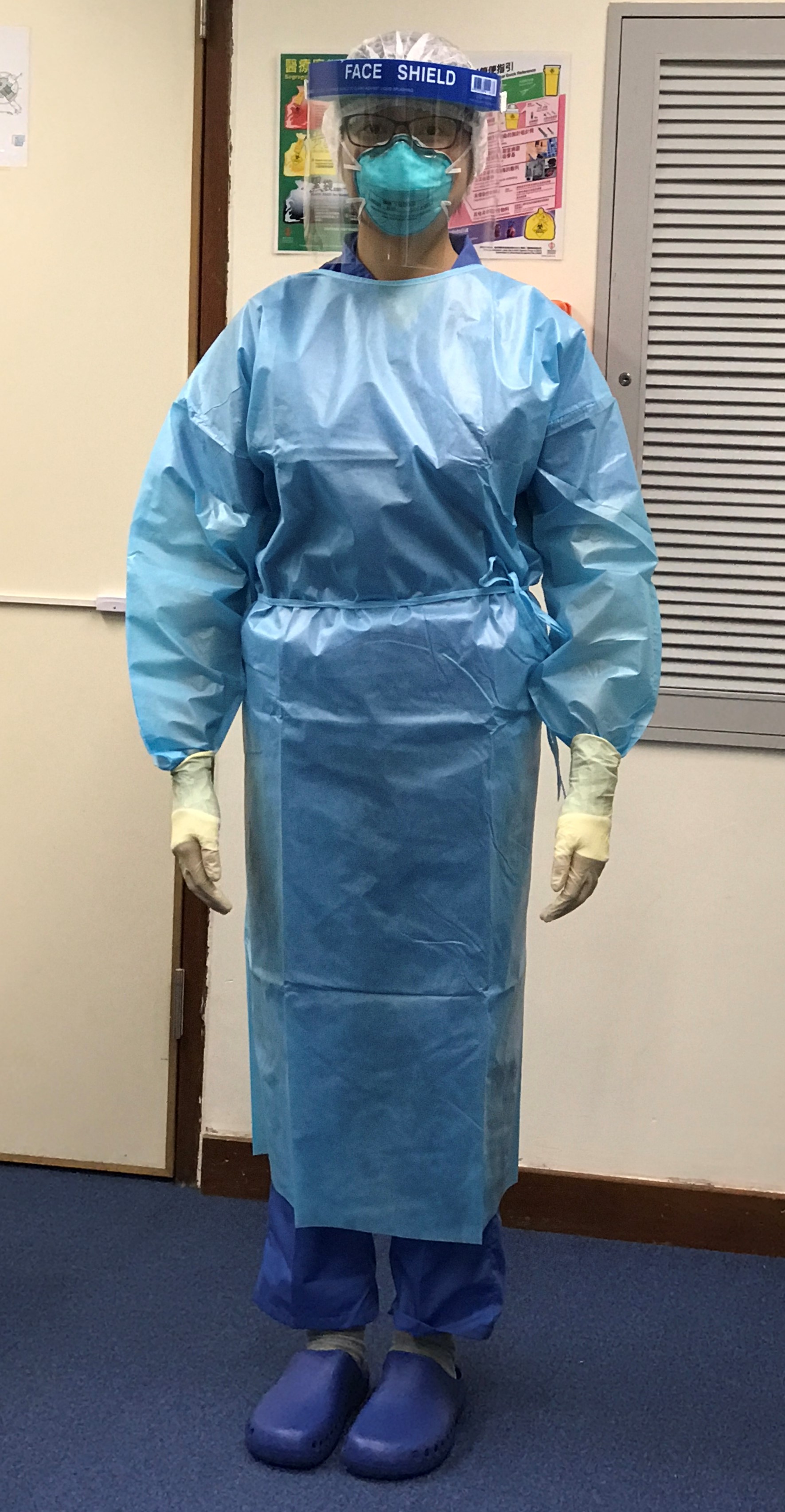

Supplement: Supplementary file 6 — Figure S6. After putting on cap and full face-shield (PPE3). (JPG 886 kb) [file 13756_2018_433_MOESM6_ESM.jpg]
